# N-acetyl Cysteine Administration Is Associated With Increased Cerebral Glucose Metabolism in Patients With Multiple Sclerosis: An Exploratory Study

**DOI:** 10.3389/fneur.2020.00088

**Published:** 2020-02-14

**Authors:** Daniel A. Monti, George Zabrecky, Thomas P. Leist, Nancy Wintering, Anthony J. Bazzan, Tingting Zhan, Andrew B. Newberg

**Affiliations:** ^1^Department of Integrative Medicine and Nutritional Sciences, Marcus Institute of Integrative Health, Thomas Jefferson University, Philadelphia, PA, United States; ^2^Department of Neurology, Thomas Jefferson University, Philadelphia, PA, United States; ^3^Division of Biostatistics, Department of Pharmacology and Experimental Therapeutics, Thomas Jefferson University, Philadelphia, PA, United States; ^4^Division of Nuclear Medicine, Department of Radiology, Thomas Jefferson University, Philadelphia, PA, United States

**Keywords:** N-acetyl cysteine, antioxidant, multiple sclerosis, cerebral glucose metabolism, positron emission tomography, cognition

## Abstract

**Background:** Multiple Sclerosis (MS) is an autoimmune disease marked by progressive neurocognitive injury. Treatment options affording neuroprotective effects remain largely experimental. The purpose of this proof of concept study was to explore the effects of N-acetyl-cysteine (NAC) on cerebral glucose metabolism (CMRGlu) and symptoms in patients with multiple sclerosis (MS).

**Methods:** Twenty-four patients with MS were randomized to either NAC plus standard of care, or standard of care only (waitlist control). The experimental group received NAC intravenously once per week and orally the other 6 days. Patients in both groups were evaluated at baseline and after 2 months (of receiving the NAC or waitlist control period) with an integrated Position Emission Tomography (PET)/ Magnetic Resonance Imaging (MRI) scanner, using 18F Fluorodeoxyglucose (FDG) to measure cerebral glucose metabolism. Following imaging evaluation at 2 months, subjects initially attributed to the standard of care arm were eligible for treatment with NAC. Clinical and symptom questionnaires were also completed initially and after 2 months.

**Results:** The FDG PET data showed significantly increased cerebral glucose metabolism in several brain regions including the caudate, inferior frontal gyrus, lateral temporal gyrus, and middle temporal gyrus (*p* < 0.05) in the MS group treated with NAC, as compared to the control group. Self-reported scores related to cognition and attention were also significantly improved in the NAC group as compared to the control group.

**Conclusions:** The results of this study suggest that NAC positively affects cerebral glucose metabolism in MS patients, which is associated with qualitative, patient reported improvements in cognition and attention. Larger scale studies may help to determine the clinical impact of NAC on measures of functioning over the course of illness, as well as the most effective dosage and dosage regimen.

## Introduction

Multiple Sclerosis (MS) is a neurological disorder associated with white and gray matter injury significantly due to autoimmune mediated inflammation processes. The course of MS can be marked be marked by discreet episodes of worsening of symptoms and/or more gradual accrual of disability across neurological and cognitive domains (1). Oxidative stress is believed to occur in association with disease processes in MS (2). Oxidative stress is classically defined as a redox imbalance in which there is an excess formation of oxidants or a decrease in amount of function of antioxidants (3). In MS, there is immunologically mediated oxidative damage, which can contribute to disruption of the blood brain barrier and neuronal and axonal injury (4, 5). High amounts of polyunsaturated fatty acids and low levels of antioxidants such as glutathione in nerve cells may render the brain vulnerable to oxidative stress (6).

Available treatments for MS are generally directed toward aspects of the immune processes thought to be central to the disease process of MS. The potential role of adjuvant therapy that may help ameliorate the effects of residual inflammation such a reduced presence of antioxidants or the consequences of prior injury causing impairment of cellular homeostasis is less well-understood. There is a need for approaches that have the potential to address such processes. Along this line of thought, it has been suggested that positively affecting the redox potential might benefit aspects neurologic functioning in the short term and may hold promise to contribute to attenuation of neurodegenerative processes that occur in MS over the longer term (7). The focus of the present proof of concept study is to evaluate possible effects of N-acetyl cysteine (NAC) in patients with MS.

Glutathione is the principle naturally occurring antioxidant in neurons which protects against oxidative damage. Importantly, glutathione levels have been found to be depleted in the brain of MS patients (8, 9). Administering glutathione directly has been shown to be difficult (10, 11) due to constraints of absorption and stability that limit bioavailability. Therefore, we explored other avenues for augmenting endogenous glutathione levels and found support in the literature that NAC might be useful in this regard. Specifically, NAC is the N-acetyl derivative of the naturally occurring amino acid, L-cysteine, and studies have suggested that it helps increase glutathione levels in the body and brain (12). In an MR spectroscopy study, increased levels of glutathione could be measured following intravenous administration of NAC (13). In subjects with increased glutathione in the blood following infusion of NAC, an increase of glutathione could also be demonstrated with MR spectroscopy of the brain.

NAC is a common over-the-counter supplement and also is available as an injectable medication. The latter form is used for prevention of liver injury following acetaminophen overdose. In animal studies, the administration of NAC has been shown to increase glutathione levels in the mouse brain (14, 15). NAC also has been shown to reduce markers of oxidative damage (16). In the exercise physiology literature, both oral and injectable NAC have been observed to reduce fatigue and improve recovery from exertion (17, 18). Importantly, while there is some absorption of NAC orally, the serum concentration of NAC after intravenous injection is much higher and was shown to increase glutathione levels in the brain (13). And while repeated infusions might result in even higher concentrations, such a regimen was deemed logistically problematic for patients with neurological conditions such as MS who already have difficulty with mobility and transportation. Thus, we developed a combined regimen of once weekly infusions of NAC augmented with daily oral intake (see below in Methods).

Our group has reported clinical and neuroimaging data using the combination of both oral and intravenous NAC in patients with Parkinson's disease (PD), another neurological condition in which oxidative damage to selected neurons is believed to play a role. We observed that in the NAC treated cohort, a significant increase of dopamine function in the primary dopamine areas of the basal ganglia could be observed coupled with improvements in standard clinical scores (19). This encouraging data led us to consider whether NAC could positively affect neurophysiology in patients with MS. While there are several human studies that have evaluated antioxidants in MS patients, none have explored the use of NAC (20).

The current study of patients with MS utilized our previously developed protocol using a combination of oral and intravenous NAC administered for 2 months to patients with MS. Participants were evaluated with fluorodeoxyglucose (FDG) positron emission tomography (PET) to assess cerebral glucose metabolism at baseline and at the end of the 2 months study period. The overarching goal was to determine if there is a discernable functional neuroimaging signal for NAC treatment in patients with MS, and to assess possible correlates in patient reported symptoms. With this goal in mind, we performed this initial study in an effort to determine feasibility of the intervention with NAC and also to detect a physiological response signal using FDG PET.

The use of functional neuroimaging with FDG PET is a key aspect of the present study and was performed with an integrated PET/MRI scanner rather than PET/CT commonly used for such studies. Most MS studies utilize structural MRI for identifying lesions and determining if they are active based on gadolinium enhancement (21). However, MRI is limited in regard to functional assessment in MS patients, and standard PET performed with PET/CT does not allow for ready visualization of white matter and of MS lesion. Using a PET/MRI has the promise of unifying the greater anatomic acuity of MRI with that of PET to better assess and localize the functional/metabolic processes. The few studies that have attempted FDG PET in MS have been non-interventional, observing abnormal metabolism in key brain areas including the frontal and parietal lobes, as well as the cerebellum (22–24). Such changes may be reflective of neuronal function and may be associated with fluctuating and persistent deficits in MS such as fatigue and cognition (25, 26). Given the potential advantage of combining FDG PET with MRI, our goal was to capture functional changes on brain PET in this initial study on MS patients treated with NAC.

## Materials and Methods

### Overview of Study

Written informed consent, approved by the Institutional Review Board of Thomas Jefferson University, was obtained from all subjects and the study was registered on clinicaltrials.gov with the following identifier: NCT03032601. Subjects were recruited from local neurology offices and also from the local community through meetings with MS support groups in the area. Subjects met 2010 diagnostic criteria for MS and fulfilled following inclusion criteria: Age 21–80 years old and on stable medication regimen for at least 1 month. Patients were excluded for the following: any intracranial abnormalities; and any medical, neurological, or psychiatric disorder that could reasonably be expected to interfere with the assessment of MS symptoms, or with the study assessments including the FDG PET imaging. Subjects that qualified for the study then underwent an initial FDG PET scan along with qualitative evaluation of their symptoms using the 36-Item Short Form Survey, the Mental Health Inventory from the Medical Outcomes Study Survey, and the Perceived Deficits Questionnaire (27, 28). While our focus was on cognition, these surveys together also assessed pain, fatigue, anxiety, and depression, as well as overall physical function.

### NAC Intervention

Subjects were randomized using a permuted block method (1:1 ratio using sealed envelopes with the allocation) to either receive intravenous/oral NAC or were placed in the waitlist control condition. For 2 months both groups continued their current standard of care MS treatment, with the experimental group receiving NAC. NAC was obtained from the Jefferson Pharmacy as Acetadote (Cumberland Pharmaceuticals). Pharmaceutical NAC is an intravenous (IV) medication most commonly used for the treatment of acetaminophen overdose. Doses of Acetadote were prepared for each patient by a trained study nurse. The dose was 50 mg/kg mixed into 200 ml of D5W infused over ~1 h 1x per week. Subjects additionally took 500 mg NAC tablets 2x per day on the days that they did not receive the IV NAC.

After ~60 days of receiving oral and IV NAC or being in the waitlist condition, subjects underwent a follow up evaluation, including repeat FDG PET imaging and clinical evaluation.

### FDG PET Imaging Procedure

FDG-PET was performed according to standard clinical protocol using the Siemens mMR PET-MRI scanner (Siemens Medical Solutions USA, Malvern, PA). Briefly, an intravenous catheter was inserted. The patient's eyes were open, ears were unoccluded, and ambient noise was kept to a minimum during the study. The patient was then injected with ~185MBq (5mCi) of FDG. Scanning was initiated 30 min after the administration of FDG. Images were obtained over a 20 min period while MR imaging was also obtained for anatomical correlation and attenuation correction. The head was fixed in place throughout the study by a head holder. At the completion of the scanning, the images were reconstructed in the transaxial planes using an iterative reconstruction process.

### Image Analysis and Statistics

FDG PET scans were analyzed utilizing the MIMNeuro software system (MIM Software Inc., Cleveland, OH). Within the x-y plane, the ROIs in the template were smaller than the actual structures they represent in order to minimize resolution-induced problems with ill-defined edges. The primary outcome measure was the cerebral glucose metabolism measure relative to the normal database that accompanies the MIMNeuro software system. This allows for a semiquantitative assessment of cerebral glucose metabolism which yields s Z score compared to a normal database that is part of the software program. The FDG PET scans are normalized to the whole brain metabolism. The change in metabolism could be compared between the pre and post-NAC scans and compared to the control group scans.

The thirty (30) cerebral glucose metabolism ROI measures, as well as the clinical measures (SF36, MFIS, MHI, and PDQ), were studied separately using linear mixed effects (LME) models with random effect of patient. The fixed effects considered include age, gender, ethnicity, treatment group (N[AC] vs. C[ontrol]), timing (pre-therapy and post-therapy), as well as the interaction of treatment group and timing. Variable selection based on second-order Akaike information criterion are performed using R (29) function “dredge” in package MuMIn (30). Correction for multiple comparisons for the analysis between the two groups was also performed on the imaging results and the clinical measures using the False Discovery Rate method.

## Results

### Study Demographics

**Figure 1:**
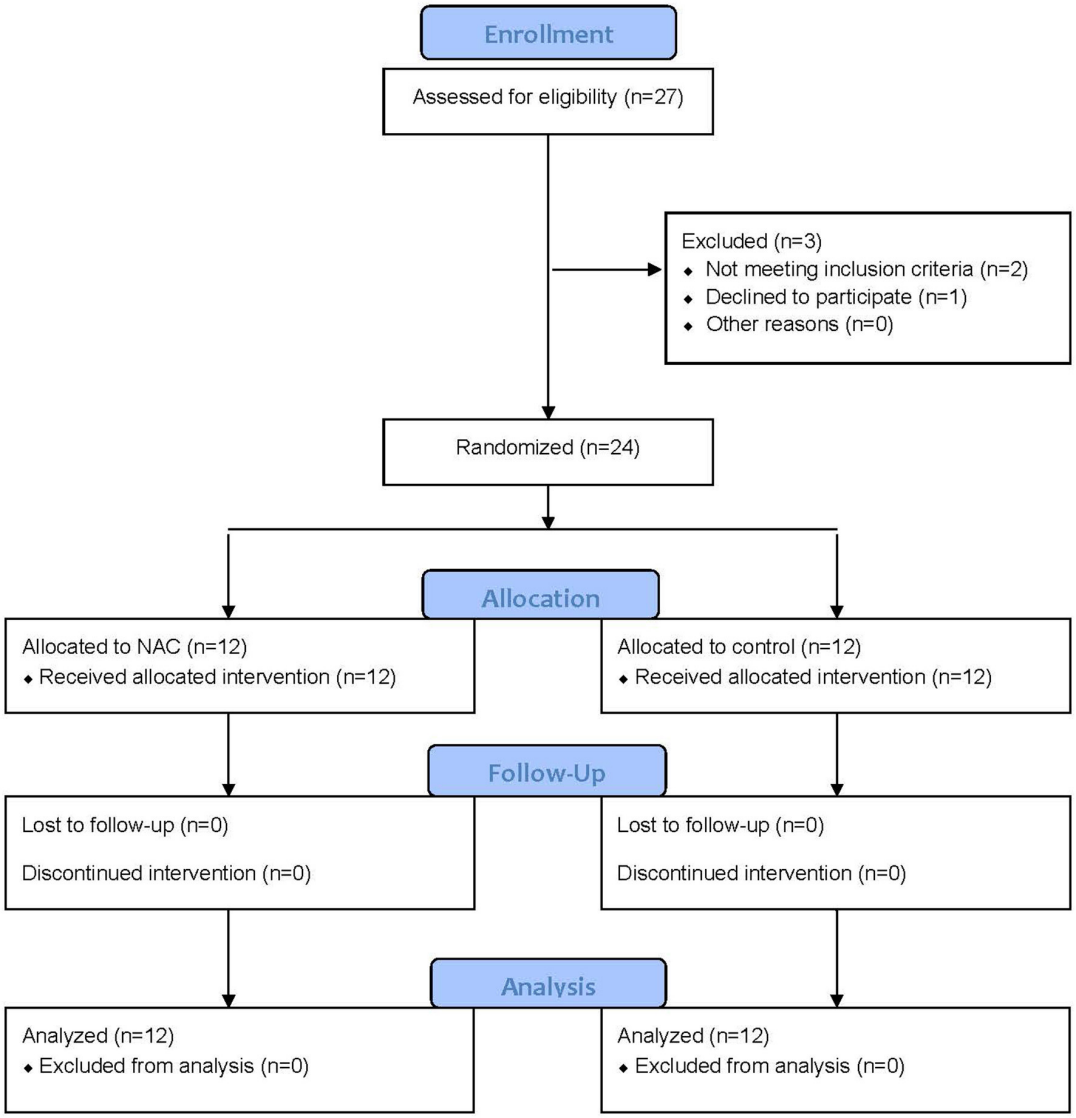
CONSORT Flow Diagram for the study participants.

For this study, 24 patients with a diagnosis of MS (18 relapsing remitting and six primary progressive) were enrolled for this preliminary assessment, with 12 patients randomized to the NAC arm (10F/2M; mean age 51.5 ± 14.4) and 12 patients to the waitlist control arm (10F/2M; mean age 53.9 ± 9.5, *p* = 0.63). All 24 patients received NAC either initially or after the 2 months observational period. Six patients reported mild gastrointestinal discomfort and five patients reported mild fatigue in proximity to infusions (see Figure 1 for the CONSORT Flow Diagram). There were no severe adverse events reported.

### Imaging and Clinical Results

**Figure 2:**
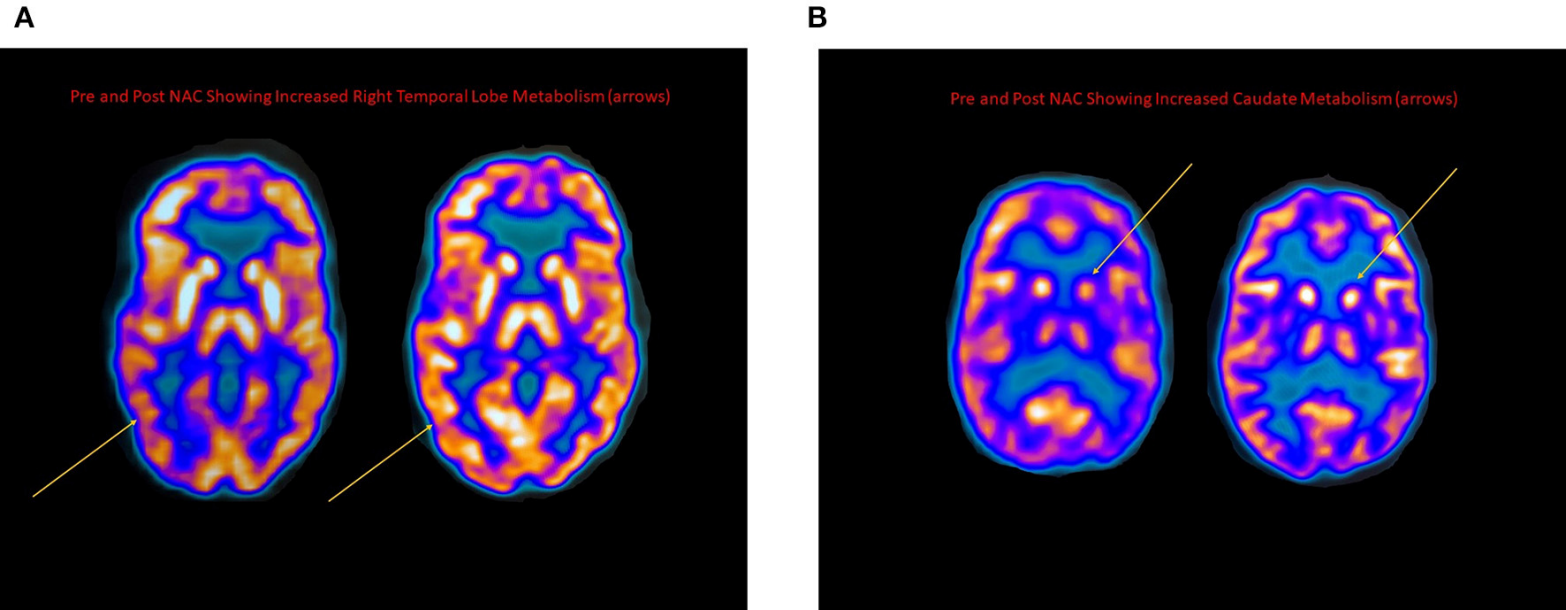
**(A)** FDG PET scans taken pre and post-NAC for an MS patient demonstrating marked improvement in the right temporal lobe cerebral glucose metabolism (arrows). **(B)** FDG PET scans taken pre and post-NAC for an MS patient demonstrating marked increase in caudate glucose metabolism (arrows).

Table 1 provides pre- to post- CMRGlu measures in each therapy group (NAC and controls) for each region of interest (ROI) that was significantly different between the two groups (*p* < 0.05) along with the corresponding *p*-value for the difference between the pre- to post- change in both the NAC group and the control group (see also Figure 2). Overall, the NAC group demonstrated significant increases (corrected for multiple comparisons) compared to controls in cerebral glucose metabolism after therapy in the lateral and middle temporal lobes, inferior frontal lobe, and caudate. In addition, the NAC group had significant improvements in self-reported levels of cognition and attention based upon their scores on the MHI cognition and PDQ attention subscores. Other subjective measures such as pain, fatigue, anxiety, depression, and overall physical function showed improvement in the NAC group but were not significantly altered in the NAC or control groups and therefore are not included in the results table.

**Table 1:** Results are provided for the ROI's with significant post- vs. pre-treatment differences in the NAC and control subjects, i.e., with the presence of treatment group and timing interaction.

**Brain structure**	**Group**	**Post- vs. pre-difference**	***p*-value**	**95% CI**
Inferior frontal gyrus	Control group	−0.060	0.993	(−0.456, 0.336)
	NAC group	0.441	0.022	(0.044, 0.837)
Lateral temporal gyrus	Control group	−0.805	0.039	(−1.579, −0.031)
	NAC group	0.854	0.025	(0.080, 1.629)
Middle temporal gyrus	Control group	−0.774	0.072	(−1.595, 0.046)
	NAC group	0.982	0.012	(0.162, 1.803)
Temporal lobe	Control group	−0.954	0.093	(−2.015, 0.106)
	NAC group	1.000	0.072	(−0.061, 2.061)
Caudate	Control group	−0.100	0.952	(−0.497, 0.297)
	NAC group	0.606	<0.001	(0.209, 1.003)
MHI cognition	Control group	−3.056	0.744	(−10.188, 4.076)
	NAC group	8.611	0.010	(1.479, 15.742)
PDQ attention	Control group	0.917	0.305	(−0.413, 2.246)
	NAC group	−1.417	0.031	(−2.746, −0.087)

*The p-values provided are for each group and the regions listed are those that were significantly different between the two groups at a p < 0.05 corrected for multiple comparisons. Similarly, the table shows the self-reported scores of cognition and attention with the presence of treatment group and timing interaction. These were the only clinical measures that were significantly improved in the NAC group compared to the control group (p < 0.05 corrected for multiple comparisons). Other measures of pain, fatigue, anxiety, depression, and overall physical function were not significantly different between the two groups*.

## Discussion

A number of studies have suggested the important role oxidative stress plays in the pathophysiology of MS, and that markers of oxidative stress are increased in MS lesions (31, 32). Glutathione is the body's natural reducing agent in neurons to help protect against oxidative damage. Glutathione levels have been shown to be reduced not only in the brain (8, 9) but also in the periphery of MS patients (33). The brain is vulnerable to high amounts of oxidative stress because of the limited about of antioxidants such as glutathione (34). Given that oxidative damage is a critical part of the MS process, the question is whether interventions designed to augment antioxidant activity will be effective for improving brain function and symptoms in these patients.

To our knowledge, this is the first study evaluating a possible effect of NAC in patients with MS, and specifically using FDG PET to measure cerebral glucose metabolism. As mentioned above, NAC is an oral over-the-counter antioxidant supplement and also is available as an injectable pharmaceutical typically used to protect the liver in cases of acetaminophen overdose. For this study, we chose a weekly injection of NAC supplemented by daily ingestion. The available literature on absorption and pharmacokinetics suggests that the injectable form is critical for achieving consistent, increased blood levels (35, 36). In addition, one MRS study suggested that there was an increase in brain glutathione levels in patients receiving an intravenous but not oral dose of NAC (13). For these reasons, in addition to our initial success with this regimen in another neurological population of patients with Parkinson's disease, we decided that the regimen of combined oral and intravenous NAC would be the best approach in terms of tolerability and logistics for patients while ensuring an adequate amount of NAC given.

The results of the present study show notable increases in cerebral glucose metabolic activity among a number of brain regions in response to NAC treatment. We should note that while we did not specifically quantify the MS lesions in these patients, clinical evaluation revealed no new lesions on any patient during the study and no obvious change in lesion volume. Future studies should explore this observation in more detail and perhaps determine whether other neurophysiological measures, such as lesion number and size as well as measures of functional connectivity, also change in a positive direction.

It is noteworthy that the temporal lobe and inferior frontal lobe had increased cerebral glucose metabolism post-treatment since these regions are known to support the cognitive processes which were perceived to be improved in the patients receiving NAC on self-reporting measures. Future studies should focus on whether these effects are long lasting with a deeper assessment of how they may correlate clinically, given that cognition is impaired in 40–70% of patients with MS (37, 38), including memory, information processing speed, attention, and executive function (39).

There are limited studies evaluating cerebral glucose metabolism in relation to cognitive impairment in MS patients. One magnetic resonance spectroscopy study suggested that abnormal cerebral glucose metabolism was correlated with abnormal values of N-acetyl-aspartate (NAA) normalized to creatine (NAA/Cr) in brain regions associated with cognition (40). Another study suggested that regional changes in frontal lobe metabolism on FDG-PET is associated with specific changes in memory in MS patient (41). It is also interesting to note that a systematic review of the effects of NAC suggest that it may generally help improve cognitive performance in a variety of conditions (42). While there were no MS studies in this meta-analysis, the suggestive efficacy signal in conditions like Alzheimer's Disease lends support to the construct of NAC improving oxidative stress pathways resulting in improved neuronal function. Thus, the findings from the current study are consistent with the above mentioned studies and suggest that NAC might improve cerebral metabolism in areas of the brain known to be associated with cognitive processing, such as the frontal and temporal lobes.

There are limitations to the current study. As an initial study exploring a response signal, we kept the sample size small, which is also limited by the cost of the PET-MRI scans pre and post-NAC. Although we included a control group, it was not a placebo group and it was not a blinded study. It is always possible that the observed changes in the NAC group could have been influenced by a placebo effect. However, given ethical concerns raised in the literature regarding giving MS patients intravenous placebo, for this initial study we opted to have a waitlist control to first see if there was a positive signal to advance to the next level of inquiry (43). Future studies comparing NAC to a placebo infusion might help to clarify this issue. It will also be essential to measure NAC or glutathione levels in the brain either through direct measurement of CSF or through an approach such as MR spectroscopy. Although this study provided patients with both oral and intravenous NAC, it is not known if the oral supplementation has significant value. This should be explored further in future studies along with an ideal time course of treatment. Specifically, we selected 2 months as an initial time period to assess for tolerability and with the goal of seeing a measurable effect on the FDG PET scans. However, most MS patients have the symptoms for years and it might be necessary to expand the duration of administration of the NAC for many months or even years in order to observe its full ability to help with the management of symptoms in patients with MS. In regard to the changes we observed in symptoms of cognition and attention, self-reported questionnaires were used rather than objective neuropsychological measures. This is important as the results reflect changes in perceived cognition which can be affected by a number of factors other than just cognition itself. For example, stress, depression, sleep, and fatigue, along with other disease related processes, can all contribute to a person's perceived, and actual, levels of cognition (44–47). Although these measures were mildly improved in the NAC group, they were not statistically significant for this sample size. Thus, we are planning to perform future studies with objective measures of cognition and attention, in addition to factoring in other disease parameters, to better determine the specific effects of NAC on cognition and attention in a larger sample of patients with MS.

Finally, this is the first study to use FDG PET to evaluate the effects of an intervention in patients with MS. It is uncertain how relevant measures of cerebral glucose metabolism are for determining physiological effects of treatments in MS patients. The focus has traditionally been on symptom improvement and prevention of new MS lesions. However, it may be that the evaluation of cerebral glucose metabolism is a valuable marker for evaluating how the brain of MS patients is responding to therapies that target neuronal function. Furthermore, data suggest that FDG PET findings can help elucidate the clinical effects of MS lesions by determining the extent to which they cause neuronal dysfunction, something that cannot be determined simply by observing the presence of the lesion. Thus, it is our hope that this methodology of using FDG PET may be useful for future studies of MS therapies in general as well as those targeting oxidative stress in particular.

Overall, the current study using FDG PET imaging suggests that NAC might positively impact cerebral glucose metabolism in MS patients, and this appears to be associated with improved symptoms related to cognition and attention. The next level of inquiry with a larger randomized, double blind, placebo controlled efficacy trial is warranted. In addition, objective measures of cognition and attention will be needed to further assess the impact of NAC on these symptoms in MS patients.

## Data Availability Statement

The datasets generated for this study are available on request to the corresponding author.

## Ethics Statement

The studies involving human participants were reviewed and approved by Thomas Jefferson University Office of Human Research. The patients/participants provided their written informed consent to participate in this study.

## Author Contributions

DM, GZ, TL, NW, AB, TZ, and AN contributed to the conception and design of the study. NW, TZ, and AN organized the database. TZ and AN performed the statistical analysis. DM and AN wrote the first draft of the manuscript. GZ, TL, NW, AB, and TZ wrote sections of the manuscript. All authors contributed to manuscript revision, read, and approved the submitted version.

### Conflict of Interest

The authors declare that the research was conducted in the absence of any commercial or financial relationships that could be construed as a potential conflict of interest.
